# Exercise training ameliorates myocardial phenotypes in heart failure with preserved ejection fraction by changing N6-methyladenosine modification in mice model

**DOI:** 10.3389/fcell.2022.954769

**Published:** 2022-09-02

**Authors:** Kai Liu, Wenhao Ju, Shengrong Ouyang, Zhuo Liu, Feng He, Jingyi hao, Hongyan Guan, Jianxin Wu

**Affiliations:** ^1^ Department of Biochemistry and Immunology, Capital Institute of Pediatrics-Peking University Teaching Hospital, Beijing, China; ^2^ Department of Biochemistry and Immunology, Capital Institute of Pediatrics, Beijing, China; ^3^ Beijing Municipal Key Laboratory of Child Development and Nutriomics, Beijing, China; ^4^ Graduate School of Peking Union Medical College, Beijing, China; ^5^ Beijing TongRen Hospital, Capital Medical University, Beijing, China

**Keywords:** HFPEF, m^6^A, apoptosis, FTO, myocardial energy metabolism

## Abstract

Heart failure with preserved ejection fraction (HFpEF) shows complicated and not clearly defined etiology and pathogenesis. Although no pharmacotherapeutics have improved the survival rate in HFpEF, exercise training has become an efficient intervention to improve functional outcomes. Here, we investigated N6-methyladenosine (m^6^A) RNA methylation modification in a “two-hit” mouse model with HFpEF and HFpEF with exercise (HFpEF + EXT). The manner of m^6^A in HFpEF and HFpEF + EXT hearts was explored *via* m^6^A-specific methylated RNA immunoprecipitation followed by high-throughput and RNA sequencing methods. A total amount of 3992 novel m^6^A peaks were spotted in HFpEF + EXT, and 426 differently methylated sites, including 371 hypermethylated and 55 hypomethylated m^6^A sites, were singled out for further analysis (fold change *>*2, *p <* 0.05). According to gene ontology (GO) and Kyoto Encyclopedia of Genes and Genomes (KEGG) pathway analyses, unique m^6^A-modified transcripts in HFpEF + EXT were associated with apoptosis-related pathway and myocardial energy metabolism. HFpEF + EXT had higher total m^6^A levels and downregulated fat mass and obesity-related (FTO) protein levels. Overexpression of FTO cancels out the benefits of exercise in HFpEF + EXT mice by promoting myocyte apoptosis, myocardial fibrosis and myocyte hypertrophy. Totally, m^6^A is a significant alternation of epitranscriptomic processes, which is also a potentially meaningful therapeutic target.

## Introduction

Heart failure (HF) with preserved ejection fraction (HFpEF) is a complicated clinical syndrome associated with poor quality of life, extensive utilization of medical resources and premature death (Dunlay et al., 2017; Mishra and Kass, 2021; Omote et al., 2022). HFpEF affects 50% of patients with HF worldwide and has been increasing in prevalence largely connected with the aging of the population (Mishra and Kass, 2021; Omote et al., 2022). HFpEF is an aging disease characterized by cardiac hypertrophy, myocardial interstitial fibers, and cardiac diastolic dysfunction (Prandi et al., 2022). Considering the complex pathogenesis of HFpEF, it is the most significant unmet medical need in cardiovascular disease (Mishra and Kass, 2021). Previous studies have suggested that HFpEF is a hemodynamic disorder characterized by hypertension, myocardial hypertrophy, myocardial fibrosis and diastolic dysfunction. However, current studies believe that HFpEF is a syndrome with abnormal function of multiple organs, including diseases caused by the comprehensive effects on the heart, lung, kidney, immunity, inflammation, metabolism and others (Figtree et al., 2021; Mishra and Kass, 2021; Omote et al., 2022; Prandi et al., 2022). Cardiac molecular and cellular mechanisms with HFpEF include cardiac hypertrophy, myocardial fibrosis, inflammation, cardiomyocyte sarcomere disfunction and mitochondrial and metabolic defects (Schiattarella et al., 2021a). However, the etiology and pathogenesis of HFpEF are unclear. At present, the effective treatment of HFpEF is very limited and cannot prevent the development of the disease. Urgent problems and effective solutions are needed.

Recent studies have shown that exercise training (EXT) is an important means of nonpharmacological intervention and prevention of cardiovascular diseases. EXT can improve exercise ability and quality of life, and is related to reducing the risk of hospitalization and cardiovascular death (Leggio et al., 2020; Jaconiano and Moreira-Gonçalves, 2022). However, its positive influence basically lacks a physiological explanation in HFpEF. In patients with heart failure with reduced ejection fraction (HFrEF), physical exercise is already a class I level A recommendation (Ho et al., 2019). Concerning HFpEF, systematic reviews and meta-analysis offer a high level of evidence that EXT is a safe and effective strategy improving upon peak VO_2_, 6 min walk, cardiorespiratory fitness, diastolic function, quality of life, and general health (Ismail et al., 2013; Dieberg et al., 2015; Fukuta et al., 2019). Another research supported that EXT significantly improves the exercise capacity and left ventricular diastolic function, indicating an improvement in ventricular stiffness and filling pressures (Edelmann et al., 2011; Fukuta et al., 2019). Nevertheless, this evidence is not consensual. Therefore, we use a new “two-hit” mouse model of HFpEF (Schiattarella et al., 2019; Schiattarella et al., 2021b), which simulates concomitant metabolic and hypertensive stress in mice to explore the effect mechanism of EXT on HFpEF.

Epigenetic regulation includes DNA methylation, histone modifications, and noncoding RNAs. Their important role in HFpEF has been widely researched, and the effect of RNA alternation on the control of gene expression in HFpEF will be clarified (Handy et al., 2011; Feil and Fraga, 2012; Poller et al., 2018; Zhang et al., 2021a; Hamdani et al., 2021). N6-methyladenosine (m^6^A) methylation is the most common internal mRNA alternation, which affects the metabolism of RNA throughout its life cycle (Wang et al., 2014a; Wang et al., 2014b; Alarcón et al., 2015). The latest evidence shows that m^6^A modification not only participates in normal biological processes, but also serves vital functions in the occurrence and progression of different heart diseases (Dorn et al., 2019; Mathiyalagan et al., 2019; Mo et al., 2019; Berulava et al., 2020; Gao et al., 2020; Krüger et al., 2020; Lin et al., 2020). But the reversal of myocardial dysfunction in HFpEF by EXT through m^6^A modification has not been studied.

The modification of m^6^A is related to different types of cardiovascular diseases, including cardiac fibrosis, cardiac hypertrophy, myocardial infarction, atherosclerosis, abdominal aortic aneurysm, heart failure and HFpEF (Dorn et al., 2019; Mathiyalagan et al., 2019; Berulava et al., 2020; Gao et al., 2020; Lin et al., 2020). Nevertheless, the transcriptome-wide distribution of m^6^A in HFpEF + EXT heart samples remains largely unknown. This research tried to clarify the m^6^A methylation profiles of heart tissue samples from HFpEF + EXT and control (HFpEF) mice and give evidence of highly diverse m^6^A-modified patterns in both groups. It is displayed that abnormal m^6^A RNA alternations in HFpEF + EXT samples are possible for modulating myocardial apoptosis and myocardial energy metabolism, close to the cardiac aging phenotype. Our outcomes offer evidence that exercise-induced m^6^A alternation is closely related to the pathogenesis of HFpEF in the new model and will promote further research on the potential drug targets of m^6^A alternation in the treatment of HFpEF.

## Materials and methods

### Animals

The contents of this study had been reviewed and approved by the ethics committee of Capital Institute of Pediatrics, whose approved license number was DWLL2019003. This study’s routines were all in accordance with corresponding ethical standards. C57BL/6J male mice were adopted for wild-type studies. All mice used in the trials were 8 weeks old (Schiattarella et al., 2019) and kept in cages at 24°C with a 12 h alternating light/dark cycle. The HFpEF model was constructed using mice with unrestricted access to HFD (High Fat Diet) food (D12492, Research Diet) and water for 16 weeks. N^ω^-nitro-l-arginine methyl ester (l-NAME) (0.5 g/L, Sigma-Aldrich) was added in the water, and the pH of the mixture was adjusted to 7.4. Mice in the control group were fed unlimited normal food and water. At the eighth week, mice began to do moderate-intensity running on a treadmill at a 10° gradient for 8 weeks (5 times a week, running for 5 days and rest for 2 days, with 5 min of running and 2 min of rest for 10 cycles, a total of 50 min of running and speed with 10 m/min). After the mice were euthanized, we collected hearts with 1.5 ml RNase-free centrifuge tubes, and stored them immediately at 4°C for 12 h. Samples were finally placed at −80°C for long-term storage to prevent RNA degradation.

### Echocardiographic assessment

At 28 weeks of age, cardiac function by echocardiography in all mice in a scrambled order was assessed with a VEVO 3100 instrument, by dedicated personnel. Anesthesia mice were induced by continuous inhalation using 2% isoflurane +2 L/min air flow rate. The probe was placed in the center of the heart to observe the short axis of the heart. Observe the long axis of the heart during the slitting and adjust the heart rate to maintain 415–460 beats/min. Coefficients gathered included: left ventricular end-diastolic diameter, left ventricular end-systolic diameter, heart rate, left ventricular shortening, LVEF, peak doppler flow velocity in early mitral valve diastolic, peak doppler flow velocity across late mitral valve diastolic. All measurements were averages of at least three beats.

### Western blotting and antibodies

Our western blotting assays were performed strictly following the standard protocols of our laboratory. Firstly, mouse heart tissues were ground using the tissue crusher with the tissue lysate, and lysates were prepared with RIPA lysis buffer from Beyotime Biotechnology, where protease inhibitor were added. Blots were screened using specific antibodies: GAPDH (5174, 1:10000; CST), Mettl3 (ab195352, 1:1000; Abcam), FTO (ab92821, 1:1000; Abcam), ALKBH5 (ab69325, 1:1000; Abcam), Mettl14 (HPA038002, 1:1000; Sigma), and m^6^A (ab208577, 1:500; Abcam).

### Histological analysis

Mouse hearts were fixed with freshly made 4% paraformaldehyde overnight, dehydrated by a dehydrator, embedded in paraffin, sliced by a paraffin slicer, 7 μm for each section, and stained with hematoxylin and eosin. Furthermore, we previously sectioned frozen heart tissue samples from mice at 7 μm slices, followed by incubation with wheat germ agglutinin (1:100) for 1 h in the dark, and finally washed three times with PBS. Cell nuclei were stained with DAPI (1:1000, 10 min) and washed thrice with PBS. Firstly, we dropped a water-soluble antifade mounting medium on the samples, then we covered the samples with glass cover slips, and finally we investigated the samples with a confocal microscope (Leica Sp8). Using the Image-Pro Plus 6.0 software to calculate the cross-sectional areas of cardiomyocytes. To detect fibrosis in the murine cardiac, we stained heart sections with the usual Masson’s trichrome procedure.

### Overexpression of fat mass and obesity-related

Empty adeno-associated virus (AAV-EV) and adeno-associated virus expressing FTO (AAV-FTO) were manufactured by Hanbio Biotechnology Ltd. (Shanghai, China). The viruses were under the control of a heart-specific *cTNT* promoter with an EGFP tag. The virus titer we used to do the experiment was approximately 1 × 10^12^ V g/ml. At 8 weeks, we injected 120 μL of AAV-EV and AAV-FTO viruses *via* tail vein for each mouse.

### m^6^A dot blot assay

We extracted total RNA from experimental mouse hearts with Trizol (Invitrogen). Total RNA was diluted to 1000 ng/μL and denaturated for 3 min at 95°C to break the RNA secondary structure into single strands. Immediately placed on ice to prevent the re-formation of RNA secondary structures. 2 μL RNA was directly dropped onto the Hybond-N+ membrane (GE Healthcare) and incubated at 80°C using an 80°C hybridizer for 1 h. Uncrosslinked RNA was washed with PBS with 0.01% Tween-20 for 5 min. We incubated membranes with anti-m^6^A antibody (1: 500 in PBS with 0.01% Tween-20) at 4°C for 12 h after blocking with 5% skim milk (in PBS with 0.01% Tween-20) for 1 h. We used horseradish peroxidase-conjugated anti-rabbit IgG secondary antibody to treat membranes for 1 h at room temperature, and rinsed 4 times for 10 min each with PBS followed by chemiluminescence development. To ensure that repeated dots contained the same amount of total RNA, methylene blue staining was utilized.

### MeRIP-seq

After three mice in HFpEF and HFpEF + EXT groups were euthanized, the total RNA samples were extracted from heart tissue specimens and quantitatively analyzed applying the NanoDrop ND-1000 (Thermo Fisher Scientific, MA, United States). Next, 20 μg total RNA was collected for interruption and purification to obtain the product after fragmentation treatment, and the Zymo RNA Clean and Concentrator-5 kit was used for the purification and recovery of the fragmented RNA. The resulting product was added with anti-m^6^A antibody (Sigma-Aldrich: ABE572), protein A-magnetic beads (Invitrogen: 10002D), protein G-magnetic beads (Invitrogen: 10004D); mixed; and incubated overnight. The mixture was subjected to magnetic separation. The supernatant was collected and added with 5× precipitation buffer and RNA enzyme inhibitor, and the mixture was reacted at 4°C for 1–3 h, washed 2–3 times with low-salt precipitation buffer, and washed 2–3 times with high-salt buffer. The RNA was extracted from chloroform lysate to obtain the purified product. Ribosomal RNA removed the obtained products, and the SMART principle synthesized the first-strand cDNA. We amplified enriched library fragments by PCR and purified the library fragments by magnetic beads with DNA to obtain an RNA methylation m^6^A detection library. Finally, we employed the bioptic qsep100 analyzer to quality check the libraries. The NovaSeq high-throughput sequencing platform and PE150 sequencing mode were adopted for sequencing.

Adapters and filters were trimed with the Cutadapt (v2.5) for sequences, and the Hisat2 aligner (v2.1.0) was adopted to align remaining reads to the human Ensemble genome GRCh38 (mouse Ensemble genome GRCm38). The exomePeak R package was adopted to identify differential m^6^A peaks. We used the clusterProfiler R package (v3.6.0) for GO and KEGG analyses. And we used the Guitar R package (v1.16.0) to visualize m^6^A-RNA-related genomic features. We subjected m^6^A peaks with *p* value <0.05 for the *de novo* motif analysis with homer (v4.10.4).

### RNA-seq

Total RNA samples were taken from heart tissue specimens after three mice in the HFpEF and HFpEF + EXT groups were euthanized. Trimming adapters and filtering for sequences was done with Cutadapt (v2.5), and the Hisat2 aligner (v2.1.0) was adopted to align the remaining reads to the human Ensemble genome GRCh38 (mouse Ensemble genome GRCm38). The feature Counts were used to determine the reads that mapped the genome (v1.6.3). Differential gene expression analysis was made with the DESeq2 R-package.

### Single-base elongation and ligation-based qPCR amplification method validation

The Single-base elongation and ligation-based qPCR amplification method (SELECT) assay was used to monitor site-specific m^6^A levels, as previously described (Xiao et al., 2018). Briefly, total RNA (2 μg) was combined with 1 μL of dNTP, 2 μL of 10 × CutSmart buffer, and 2 μL of every 400 nmol/L up and down primer (Table.1). RNA free water to the final volume 17 μL. The combination of RNA and primers was annealed at the following temperatures: 90°C for 1 min, 80°C for 1 min, 70°C for 1 min, 60°C for 1 min, 50°C for 1 min, and 40°C for 6 min. Then add 3 μL solution with 0.01 U DNA polymerase, 0.5 U SplintR ligase and 10 nmol ATP. After incubation at 40°C for 20 min and 80°C for 20 min, template abundance was quantified by taking 2 μL of reaction solution for real-time qPCR analysis.

**TABLE 1:** The gene-specific SELECT-PCR primers used were as follows.

Gene	Forward and reverse primer
Fas_	tag​cca​gta​ccg​tag​tgc​gtg​ACA​GCC​CAG​ATC​CAC​AGC​ATG
	5phos/CTGCAGCAAGGGAAAACAGCcagaggctgagtcgctgcat
Capn2	tag​cca​gta​ccg​tag​tgc​gtg​CCC​CTC​GGC​CGC​TTC​GCG​G
	5phos/CCTTGGCCAGCTTTATCGCGATGCcagaggctgagtcgctgcat
Casp12	tag​cca​gta​ccg​tag​tgc​gtg​GTC​ATC​AAA​AAC​CCC​ATC​CAG​CAT​G
	5phos/CCTTGGCCAAACCTTTGATCTcagaggctgagtcgctgcat
Mef2a	tag​cca​gta​ccg​tag​tgc​gtg​GCT​GCT​GGA​GCT​GCT​CAG​ACT​G
	5phos/CCACAGGGGAGCGCCCCcagaggctgagtcgctgcat
Casp7	tag​cca​gta​ccg​tag​tgc​gtg​GAG​CCT​GGC​TCC​AAT​CAC​CAT​AG
	5phos/CCATGGTTCTAGTCTCTAGAAGGCTGCcagaggctgagtcgctgcat
Jun	tag​cca​gta​ccg​tag​tgc​gtg​CCC​GGC​CAC​TTG​TTA​CCG​G
	5phos/CCTCTGGGTCAGGAAAGTTGCTGcagaggctgagtcgctgcat
Pdia3	tag​cca​gta​ccg​tag​tgc​gtg​TGG​TTT​TGC​CTT​CTC​TGG​TGT​AAG​AG
	5phos/CCTTTTATAAAGTGGTGCATTTGGCTcagaggctgagtcgctgcat
Tnnt2	tag​cca​gta​ccg​tag​tgc​gtg​GAC​TGC​ACA​CAG​GTC​TTG​AGG​TAT​CTG
	5phos/TCAGCCTCAGCAGGGACTGGCcagaggctgagtcgctgcat
Adrb1	tag​cca​gta​ccg​tag​tgc​gtg​CGT​CCA​GGC​TCG​AAT​CGC​TG
	5phos/CCACAGTGGTTGTCCCGCCTcagaggctgagtcgctgcat
Agtr1a	tag​cca​gta​ccg​tag​tgc​gtg​GTC​CTT​TGG​TCG​TGA​GCC​ATT​TAG
	5phos/CCGATGCTGCCCTGGTTTCTcagaggctgagtcgctgcat

### Real-time PCR (RT-PCR)

We picked 10 genes with differentially methylated sites based on MeRIP-seq, and tested these genes with Real-time PCR using SYBR-green and standard amplification protocols. The ΔΔCt technique was used to calculate expression levels. Table 2 shows the primer sequences.

**TABLE 2:** The gene-specific PCR primers used were as follows.

Gene	Forward and reverse primer
Fas	F: 5′-AAC​ATG​GAA​CCC​TTG​AGC​CA-3′
	R:5′ AGG​CGA​TTT​CTG​GGA​CTT​TGT 3′
Caspase-12	F:5′ -TGC​GAG​TTT​CAT​CCT​GAA​CAA​GGC​TG 3′
	R:5′ -AAC​ACC​AGG​AAT​GTG​CTG​TCT​GAG​GAC​T 3′
Mef2a	F:5′ GGTGGTGGCAGTCTTG 3′
	R:5′ TATCCTTTGGGCATTCA 3′
Jun	F:5′ CGC​ACG​CTC​CTA​AAC​AAA​CT 3′
	R:5′ GTC​ATA​GAA​CGG​TCC​GTC​ACT​T 3′
Tnnt2	F:5′ GCCCACATGCCTGCTT 3′
	R:5′ CACCTCCTCGGCGTCA 3′
Agtr1a	F:5′ CTG​AGG​TGG​AGT​GAC​AGG​TT 3′
	R:5′ TTT​GGT​CGT​GAG​CCA​TTT​A 3′

### Exercise exhaustion test

After 3 days of treadmill adaptation, experimental mice were tested for fatigue. Mice ran upward (20°) on a treadmill at a warm-up rate of 5 m/min for 4 min, which was grown to 14 m/min for 2 min. Subsequently, the speed was grown by 2 m/min every 2 min until the mice were exhausted. Exhaustion was defined as the inability of the mouse to resume running within 10 s of direct contact with the electrical stimulus net. While measuring the running time, the running distance was calculated.

### Transferase-mediated dUTP nick-end labeling

TUNEL experiments were made with the *In Situ* Cell Death Detection Kit (Cat. No.11684795910, Roche). The tissue section was fixed with fixation solution (4% paraformaldehyde in PBS, pH 7.4, freshly prepared) for 20 min at +15°C to +25°C and washed for 30 min with PBS. The 2-minute incubation of slides was made in permeabilization solution (0.1% Triton X-100, 0.1% sodium citrate, freshly prepared) on ice. Next, slides were added with 50 μL TUNEL reaction mixture and incubated in a humidified atmosphere for 60 min at +37°C in the dark. Finally, samples were directly analyzed under a confocal microscope.

### Transmission electron microscopy

Mouse hearts were fixed in 2.5% cacodylate buffer with glutaraldehyde, embedded with 2% agarose, fixed in 1% osmium tetroxide buffer, stained in 2% uranyl acetate, dehydrated with ethanol, embedded in resin, sliced by an ultrafine slicing machine, and stained with 2% uranyl acetate and lead citrate. Images for experimental use were obtained on the FEI Tecnai G2 Spirit electron microscope equipped with LaB6.

### Statistical analysis

All data for all experiments are from three or more independent experimental manipulations and are shown as mean ± SEM. The GraphPad Prism 6.0 program was used to conduct the statistical analysis. Differences with two groups were used two tailed unpaired Student’s t-test analysis and three or more groups were analyzed with a one- or two-way analysis of variance. Differences with a significance level of *p* < 0.05 were of statistical significance.

## Results

### Exercise reverses heart failure with preserved ejection fraction phenotypes by suppressing myocardial fibrosis and improving cardiac dysfunction with the changes in global m^6^A levels

**FIGURE 1:**
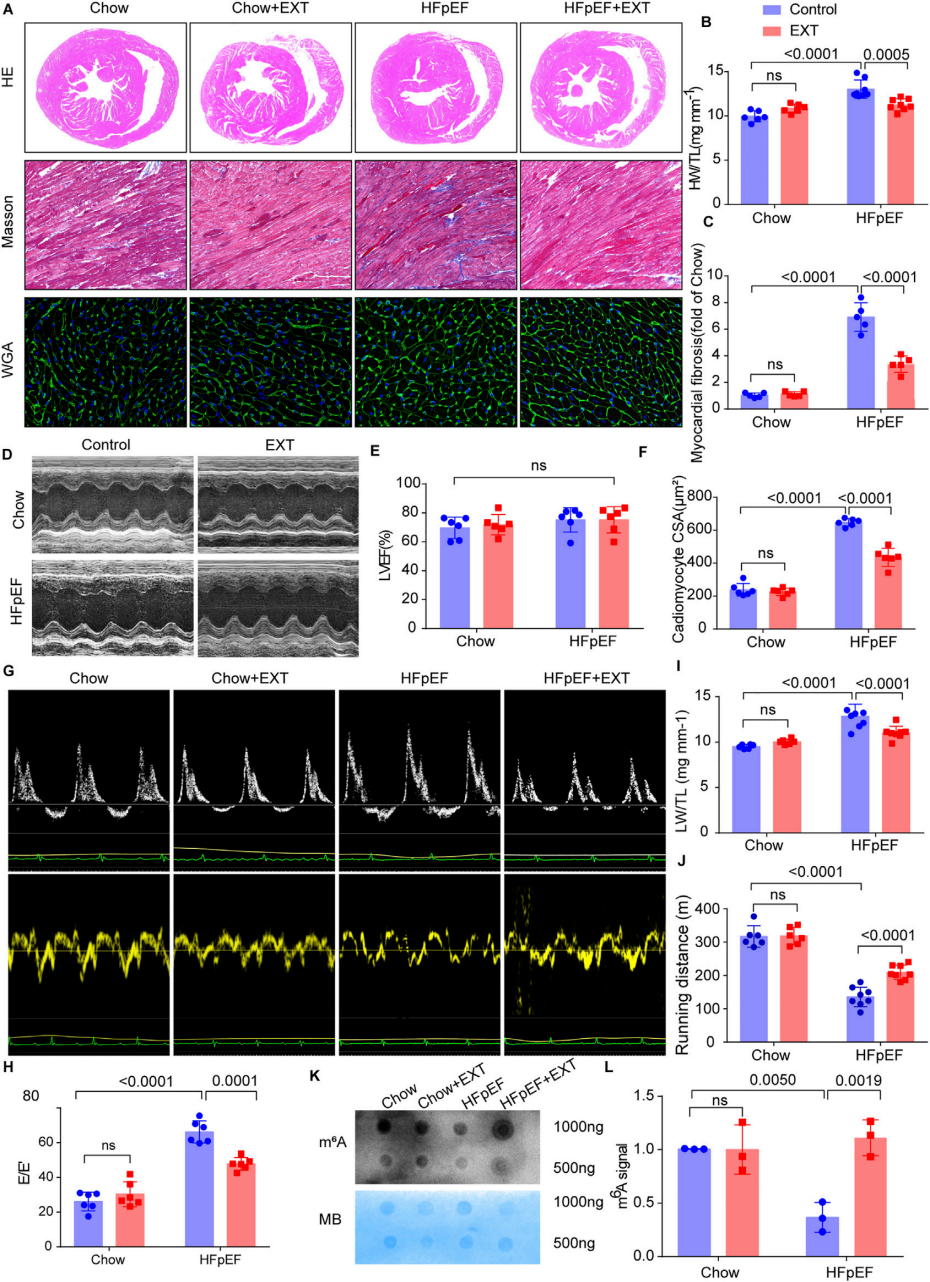
Exercise training reverses HFpEF phenotypes by suppressing myocardial fibrosis and improving cardiac dysfunction with the changes in global m^6^A levels. **(A)** Gross morphology of hearts stained with HE, cardiac fibrosis stained with Masson, and cardiomyocyte area stained with wheat germ agglutinin (WGA). Scale bar = 1 mm with HE, 100 μm with Masson, and 50 μm with WGA. **(B)** Proportion of heart weight to tibia length (*n* = 6 mice per group). **(C)** Quantitative analysis of cardiac fibrosis (*n* = 6 mice per group). **(D)** Representative M-mode echocardiography images. **(E)** Left ventricular ejection fraction (LVEF) (*n* = 6 mice per group). **(F)** Quantitative analysis of cardiomyocyte area (*n* = 6 mice per group). **(G,H)** Representative Doppler echocardiography images and E/E′ ratio (*n* = 6 mice per group). **(I)** Ratio of lung weight to tibia length (*n* = 6 mice per group). **(J)** Running distance during exercise exhaustion test (*n* = 6 mice per group). **(K,L)** Representative dot blot showing global m^6^A modification levels in hearts and methylene blue (MB) staining and quantification of global m^6^A modification levels (*n* = 3 mice per group). Data are mean ± SEM, with all individual data points plotted. Two-way ANOVA and Tukey’s multiple comparison test were used. Numbers above square brackets show significant *p* values.

In our study, HFpEF + EXT mice evidently ameliorated myocardial fibrosis and myocardial hypertrophy compared with the control (Figures 1A,B) and had evidently decreased heart weight to tibia length proportions (HW/TL, *n* = 6, *p* = 0.0005; Figure 1B). Furthermore, EXT clearly ameliorated myocardial interstitial fibrosis (*n* = 6, *p* < 0.0001; Figures 1A,C) and markedly reduced the cardiomyocyte cross-sectional region (*n* = 6, *p* < 0.0001; Figures 1A,F). In order to prove the improvement of cardiac function in mice, we performed serial echocardiography in HFpEF + EXT mice. Longitudinal echocardiographic evaluation revealed the persistent preservation of LVEF in all groups (*n* = 6, ns; Figures 1D,E), coupled with significant alterations in degrees of diastolic dysfunction and left ventricular filling pressure in mice exposed to the EXT (*n* = 6, *p* < 0.0001; Figures 1G,H). Consistent with the increase of filling pressures reported in the literature, mice exposed to EXT specifically exhibited a significant decrease in lung weight to tibia length ratios (LW/TL, *n* = 6, *p* < 0.0001; Figure 1I), indicating improvement in pulmonary function. Furthermore, the running distance was further in HFpEF + EXT mice compared with HFpEF mice (*n* = 6, *p* < 0.0001; Figure 1J). Overall, these data demonstrated that EXT reversed HFpEF phenotypes by suppressing myocardial fibrosis and improving cardiac dysfunction.

The global m^6^A level is an important feature of m^6^A modification. Therefore, the global m^6^A levels of mouse hearts in different groups were detected. Dot blot examination of cardiac samples from separate groups revealed that HFpEF + EXT mice had higher total m^6^A levels than HFpEF mice. (Figures 1K,L, *p =* 0.0019). Next, we performed transcriptome-wide MeRIP-seq and mRNA-Seq to analyze the effect of EXT on the m^6^A methylation of HFpEF.

### Exercise training demonstrates differential m^6^A modification patterns in heart failure with preserved ejection fraction mouse hearts

**FIGURE 2:**
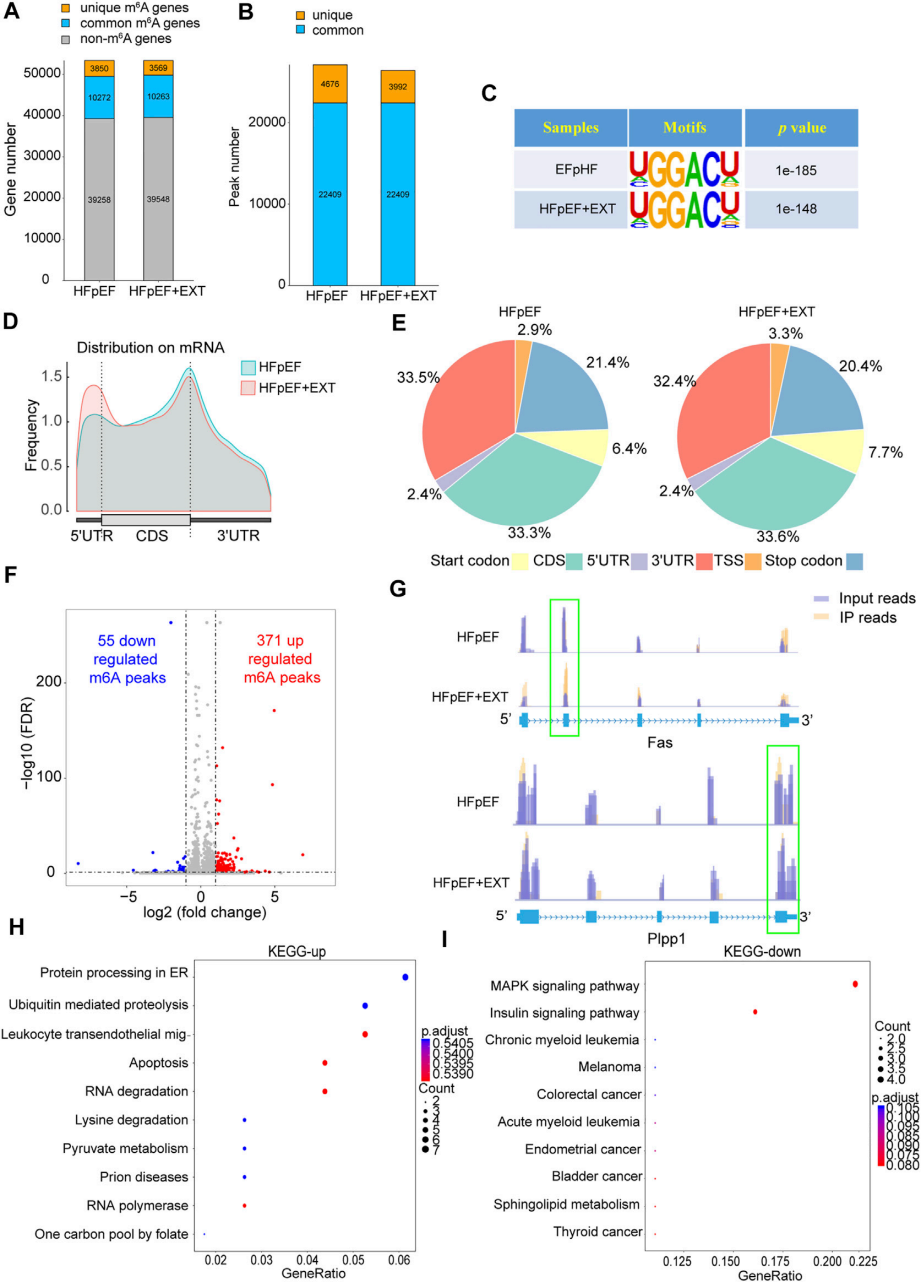
Exercise training demonstrates differential m^6^A modification patterns in HFpEF mouse hearts. **(A,B)** Summary of genes with identified m6A modification present at m^6^A-seq. **(C)** Sequence logo standing for the consensus motif identified by Discriminative Regular Expression Motif Elicitation in the two groups.**(D)** Distribution of N6-methyladenosine peaks across the length of mRNAs in two groups.**(E)** Venn diagram showing distribution of m^6^A peaks in the indicated regions in HFpEF and HFpEF + EXT groups.**(F)** Identification of 371 hypermethylated and 55 hypomethylated m^6^A peaks.**(G)** N6-methyladenosine abundance in Fas and Plpp1 mRNA transcripts in HFpEF and HFpEF + EXT samples **(H,I)** The top 10 enriched pathways terms from MeRIR-seq for upregulated and downregulated m^6^A peaks. Leukocyte transendothelial mig-: Leukocyte transendothelial migration.

HFpEF + EXT mouse hearts had specific m^6^A alternation patterns which were different from those of HFpEF mouse heart samples. We found 26401 m^6^A peaks, corresponding to 13832 gene transcripts in the HFpEF + EXT group by the model-based analysis through exomePeak v2.13.2 (Figures 2A,B). In the HFpEF group, 27085 m^6^A peaks, representing 14122 gene transcripts (Figures 2A,B), were confirmed. Compared with the HFpEF group, HFpEF + EXT hearts had 3992 novel peaks, and 4676 peaks were absent. This result indicated that the global m^6^A alternation types in the HFpEF + EXT group were different from those in the HFpEF group (Figure 2B). We identified a map of m^6^A methylation using the MEME-ChIP software and confirmed the top consensus motif in m^6^A peaks as GGACU (Figure 2C). The GGACU was parallel to the previously confirmed RRACH motif (where R = G or A; A = m^6^A, and H = U, A, or C) (Dominissini et al., 2012; Meyer et al., 2012).

We found that m^6^A peak in the HEpEF + EXT group was mainly increased in the 5′ untranslated region (5′ UTR) and decreased in the 3′ untranslated region (3′ UTR) compared with the HFpEF group. (Figure 2D). According to their precise positions in RNA transcripts, we categorized the total and unique m^6^A peaks in HFpEF + EXT and HFpEF whole-transcriptome data into 5′ UTR, transcription start site region, coding sequence (CDS), stop codon, and 3′ UTR. Our results showed that the enrichment of m^6^A peak was mainly concentrated in the region near CDS, 3′ UTR, and stop codon vicinity regions (Figure 2E), which was similar to previous m^6^A-seq results (Dominissini et al., 2012). HFpEF + EXT-specific m^6^A peak distributional patterns showed a different style from HFpEF-specific peaks, and which was with a relative increase in CDS, TSS and Start codon regions (33.3% vs. 33.6%, 3.3% vs 2.9%; 7.7% vs. 6.4%; Figure 2E) and a relative decrease in Stop codon and 3′ UTR, (20.4% vs. 21.4%, 32.4% vs. 33.5%; Figure 2E).

### Kyoto encyclopedia of genes and genomes pathways enriched for differential m^6^A methylation transcripts in heart failure with preserved ejection fraction + Exercise training group are mainly concentrated in apoptosis, RNA degradation, and MAPK signaling pathways

Differential methylation transcripts were identified and analyzed using GO and KEGG pathway analyses. By comparing the abundance of m^6^A peaks between HFpEF + EXT and HFpEF samples, we found that there were 22409 m^6^A peaks in both samples. We detected a total of 426 differentially methylated sites and took them as the research target for further research. Among these differentially methylated sites, 371 hypermethylated and 55 hypomethylated m^6^A sites were observed in the HFpEF + EXT group compared with HFpEF (fold change [FC] *>* 2, *p <* 0.05; Figure 2F). When we analyzed the differentially methylated places in both groups using the Integrative Genomics Viewer software, we found that there was significantly altered intensity in the HFpEF + EXT group. Figure 2G showed the representative m^6^A-methylated mRNA peaks in the Fas/APO-1 receptors (*Fas*) and phospholipid phosphatase-1 (*Plpp1*) genes, which were the sites with reduced and grown m^6^A levels, respectively.

In order to explore the potential biological significance of exercise-related m^6^A methylation changes in HFpEF, we performed the GO analysis of differentially methylated RNAs. Compared with those in HFpEF mice, our results revealed that hypermethylated and hypomethylated RNAs in HFpEF + EXT mice were especially related to energy metabolism terms, e.g., regulation of carbohydrate catalog, regulation of glycolytic process and carbohydrate catabolic process, suggesting that differentially methylated RNAs were mainly concentrated in energy metabolism (Supplementary Figure S2). Furthermore, the KEGG pathway analysis of differentially methylated RNAs in HFpEF + EXT mice were predominantly concentrated in myocardial cell death and myocardial energy metabolism, e.g., apoptosis, pyruvate metabolism, MAPK signaling pathway, insulin signaling pathway and sphingolipid metabolism pathway, which are mainly and strongly connected with myocardial apoptosis (Figures 2H,I). In conclusion, our findings suggested that these differentially methylated RNAs might take part in the myocardial energy metabolism and apoptosis pathway.

Overall, the transcripts of HFpEF + EXT-specific m^6^A peaks were mainly concentrated on myocardial cell death and myocardial energy metabolism, which were the main pathological features of HFpEF.

### Conjoint analysis of m^6^A-seq and RNA-Seq data shows that specific m^6^A-modified transcripts were extremely correlated with the myocardial remodeling pathological characteristics of heart failure with preserved ejection fraction

**FIGURE 3:**
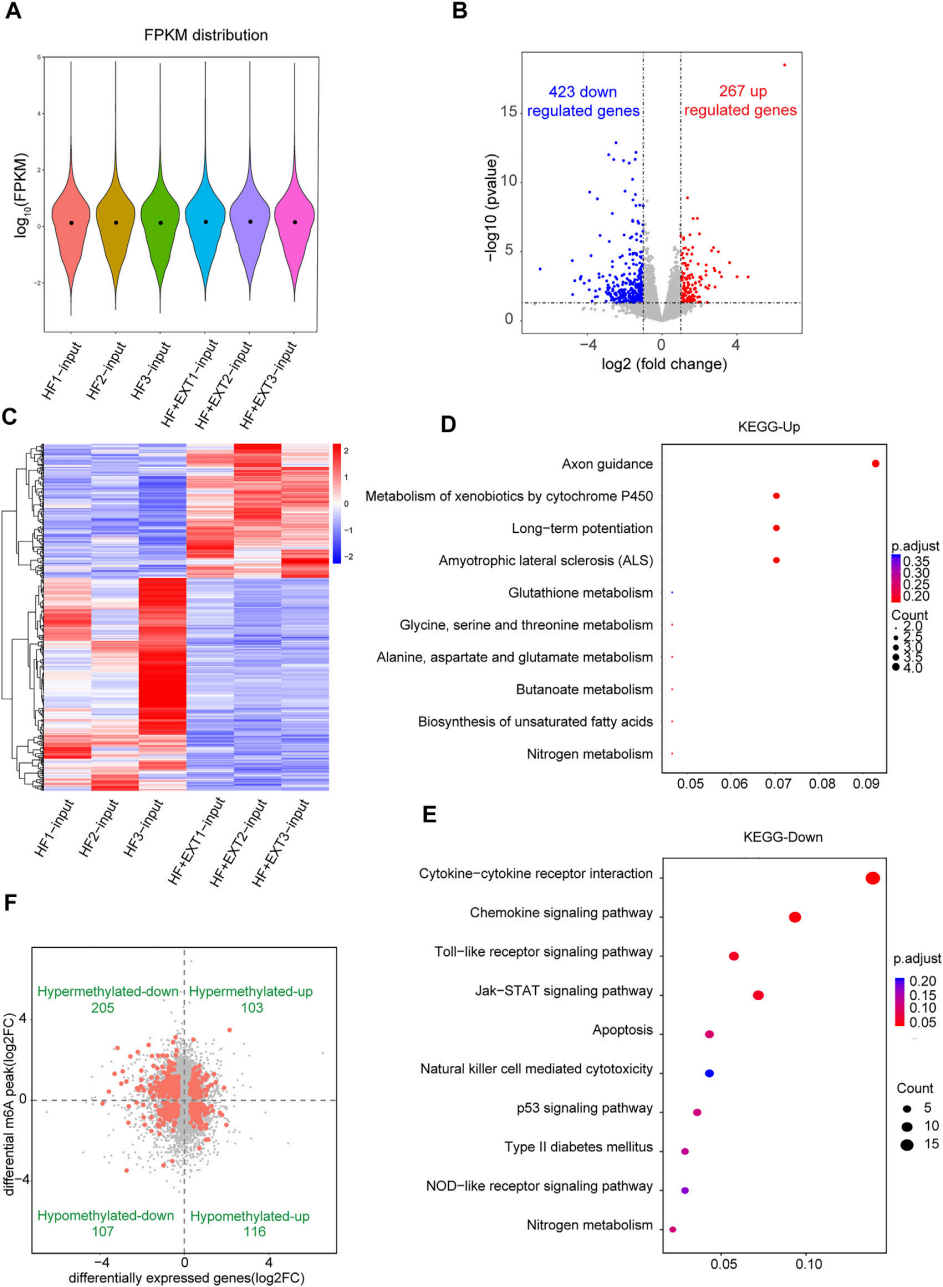
Combined analysis of RNA-seq and MeRIP-seq data comparing HFpEF with HFpEF + EXT heart samples. **(A)** FPKM distribution of two groups. **(B)** Volcano plots showing different expression mRNAs between HFpEF and HFpEF + RE samples (fold change >2 and *p* < 0.05). **(C)** Clustering analysis of differentially expressed mRNAs. **(D,E)** The top 10 enriched KEGG pathways of differentially mRNA for upregulated and downregulated mRNA. **(F)** The distribution of transcripts with significantly changed in m^6^A-modification level and corresponding mRNA expression (*p* < 0.05) by four-quadrant graph. Red dots represent significant differences in RNA expression and m^6^A modification, and gray dots do not meet the conditions.

The reads per kilobase per million mapped reads (FRKM distribution) indicated no difference in gene expression between samples (Figure 3A). RNA-Seq results indicated that 690 mRNAs, including 423 downregulated and 267 upregulated mRNAs, in HFpEF + EXT samples were significantly dysregulated compared with those in HFpEF samples (FC > 2, *p* < 0.05; Figure 3B). The tendency of differing gene expression between the groups was contemporaneous among individual samples within every group, according to further hierarchical clustering analysis of RNA-Seq data. (*n* = 3 per group, Figure 3C). Importantly, GO and KEGG pathway analysis displayed that differentially expressed genes were closely connected with apoptosis pathway and myocardial energy metabolism pathway (Figures 3D,E; Supplementary Figure S3), and this finding was coincident with the involvement in myocardial remodeling pathology (Dunlay et al., 2017; Figtree et al., 2021; Mishra and Kass, 2021; Omote et al., 2022; Prandi et al., 2022).

**FIGURE 4:**
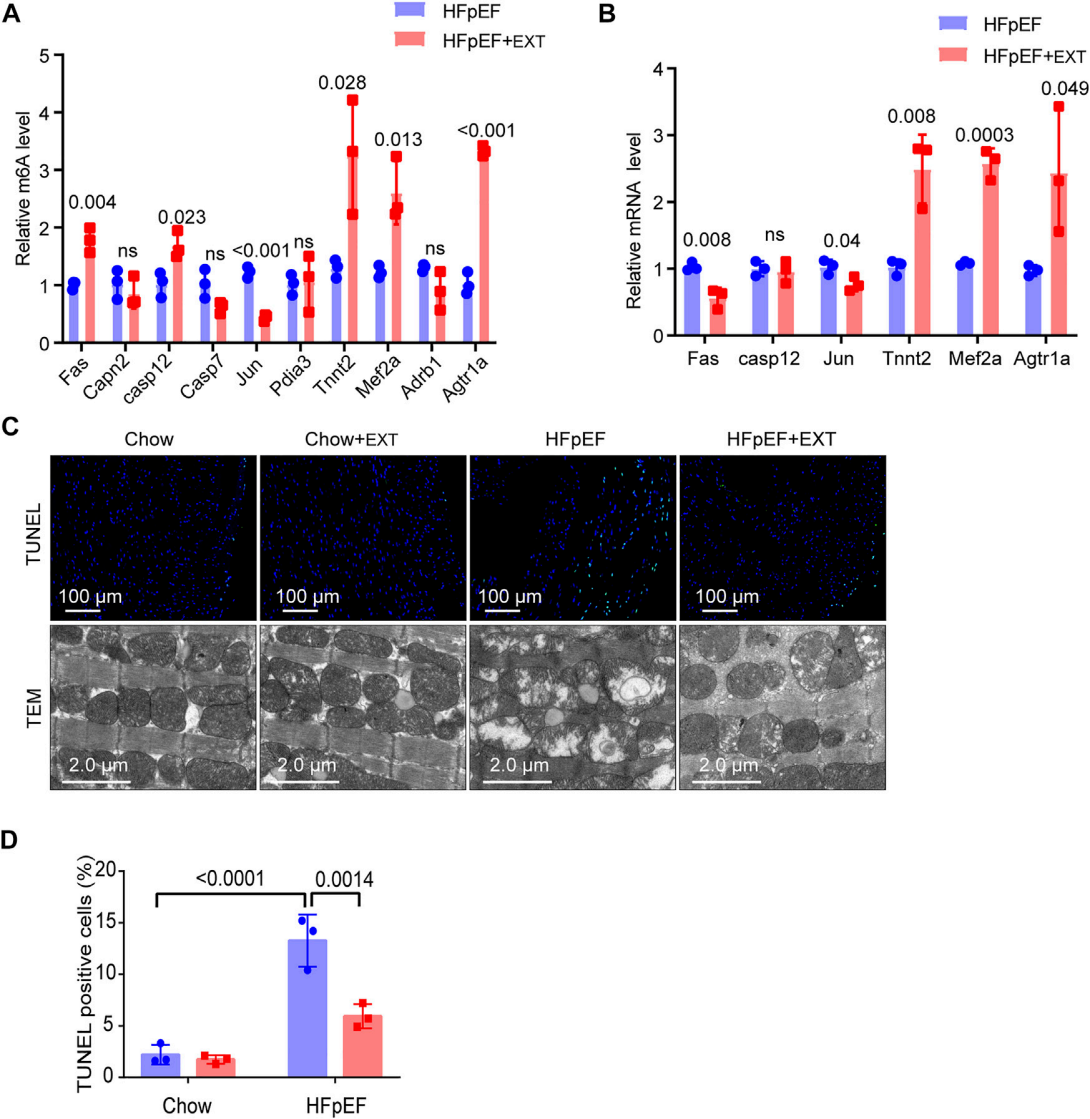
Identification of the downstream target genes and mechanisms of exercise improvement of HFpEF. **(A)** Single-base elongation and ligation-based qPCR amplification method (SELECT) validation of m^6^A level changes of ten related genes. **(B)** The relative mRNA levels of six genes determined by real-time PCR in HFpEF and HFpEF + EXT samples. *n* = 3 mice per group. Data are presented as mean ± SEM. Multiple unpaired t tests were used. **(C,D)** Representative images of TUNEL and TEM and quantitative analysis of TUNEL-positive cells. *n* = 3 mice per group. Data are presented as mean ± SEM. Two-way ANOVA and Tukey’s multiple comparison test were used. Numbers above square brackets display great *p* values.

In order to further study the target genes modified by m^6^A, target genes were idenfieid by the conjoint analysis of m^6^A-seq and RNA-Seq data. Through bioinformatics analysis, we confirmed 308 hypermethylated m^6^A peaks in mRNA transcripts, of which 103 and 205 were significantly upregulated and downregulated respectively (Figure 3F). Furthermore, 223 hypomethylated m^6^A peaks were confirmed in mRNA transcripts, of which 116 and 107 peaks were downregulated and upregulated, respectively (Figure 3F). Furthermore, the integrated analysis of MeRIP-seq and RNA-Seq data and KEGG pathway analysis showed that specific m^6^A-modified transcripts were closely associated with myocardial energy metabolism, especially myocardial apoptosis. Hence, we identified genes that were critical to these processes and further validated them, including *Fas, Capn2, Casp12, Casp7, Jun, Pdia3, Tnnt2, Mef2a, Adrb1* and *Agtr1a* (Figure 4A; Table 3).

**TABLE 3:** The ranking of the top 10 genes in each quadrant graph.

Gene name	Pattern	Chromosome	m^6^A level change				mRNA level change			
			Peak region	Peak start	Peak end	fold_enrchment	diff. lg. p	Strand	log2FC	*p* value
Casp12	Hyper-down	9	CDS	5345459	5346656	1.72	−9.81	+	−1.27477	0.000628688
Capn2	Hyper-down	1	five_prime_utr	182517278	182517608	22.6	−6.37	−	−0.30561	0.032386574
Fas	Hyper-down	19	CDS	34290658	34309091	1.79	−17.3	+	−1.03262	0.002731876
Casp7	Hypo-down	19	three_prime_utr	56441600	56441809	3.37	−2.04	+	−0.53649	0.022176301
Jun	Hypo-down	4	CDS	95049867	95052222	34.9	−1.45	−	−0.98579	5.99E-08
Pdia3	Hypo-down	2	three_prime_utr	121435993	121437910	3.93	−4.47	+	−0.50359	0.005265144
Tnnt2	Hyper-up	1	exon	135836353	135836534	2.41	−8.85	+	0.730282	0.001676025
Mef2a	Hyper-up	7	five_prime_utr	67349856	67372858	2.8	−5.61	−	0.439723	0.005269679
Adrb1	Hypo-up	19	CDS	56723142	56724091	72.7	−5.32	+	0.530445	0.01782968
Agtr1a	Hypo-up	13	CDS	30381099	30382388	14.5	−1.97	+	0.5337	0.033615285

We used SELECT-PCR to validate the m6A levels of those different critical genes *Fas, Capn2, Casp12, Casp7, Jun, Pdia3, Tnnt2, Mef2a, Adrb1* and *Agtr1a*, which were associated with myocardial apoptosis and myocardial energy metabolism. We found that the m^6^A methylation levels of *Fas*, *Casp12, Tnnt2, Mef2a* and *Agtr1a* were significantly increased and the m^6^A methylation of *Jun* was significantly decreased (Figure 4A). Furthermore, the mRNA levels of *Fas, Casp12, Jun, Tnnt2, Mef2a* and *Agtr1a* were measured in HFpEF + EXT and HFpEF hearts (Figure 4B), and results showed that *Jun, Tnnt2, Mef2a* and *Agtr1a* had similar mRNA expression tendencies in keeping with their m^6^A methylation levels, but *Fas* had opposite mRNA expression tendencies in keeping with their m^6^A methylation levels (Figure 4A).

### Exercise training ameliorates myocardial apoptosis in heart failure with preserved ejection fraction by decreasing apoptosis pathway and increasing myocardial energy metabolism

Apoptosis is one of the most important characteristics of pathological cardiac remodeling, and exercise training is an effective method to reduce cardiomyocyte apoptosis. To investigate the effect of exercise on cardiomyocyte apoptosis in the HFpEF heart, we used terminal deoxynucleotidyl transferase-mediated dUTP nick-end labeling (TUNEL) to stain cardiac pathological sections. The representative fluorescence photos of TUNEL positive nuclei in the HFpEF heart after exercise are shown in Figure 4C. In the myocardium of HFpEF mice, the proportion of TUNEL positive cardiomyocytes was greatly higher than that of control mice, while a smaller number of TUNEL positive cells could be detected in the myocardium of control or HFpEF mice after exercise (Figures 4C,D).

In order to verify whether exercise can exert its cardioprotective function by maintaining the integrity and function of mitochondria, we measured and detected the mitochondrial structure of the heart of control group and HFpEF mice with or without exercise using an electron microscope. The ultrastructure of heart sections of control group and HFpEF mice with or without exercise was analyzed by an electron microscope to determine the integrity of mitochondria (Figure 4C). In the normal mouse myocardium, mitochondria were well aligned between the longitudinally oriented myocardial myofibrils, and no difference was observed after exercise. However, the abundance of mitochondria in HFpEF appeared to be significantly altered, and the arrangement of mitochondria in HFpEF mice was disorganized, and the mitochondria were swollen and aggregated, and cristae were slightly loosened (Figure 4C). After 2 months of exercise, there was evidence that damage to cardiac mitochondria and disrupted cristae were restored in HFpEF + EXT hearts (Figure 4C).

### Overexpression of fat mass and obesity-related cancels out the benefits of exercise in heart failure with preserved ejection fraction + Exercise training mice by promoting myocyte apoptosis, myocardial fibrosis and myocyte hypertrophy

**FIGURE 5:**
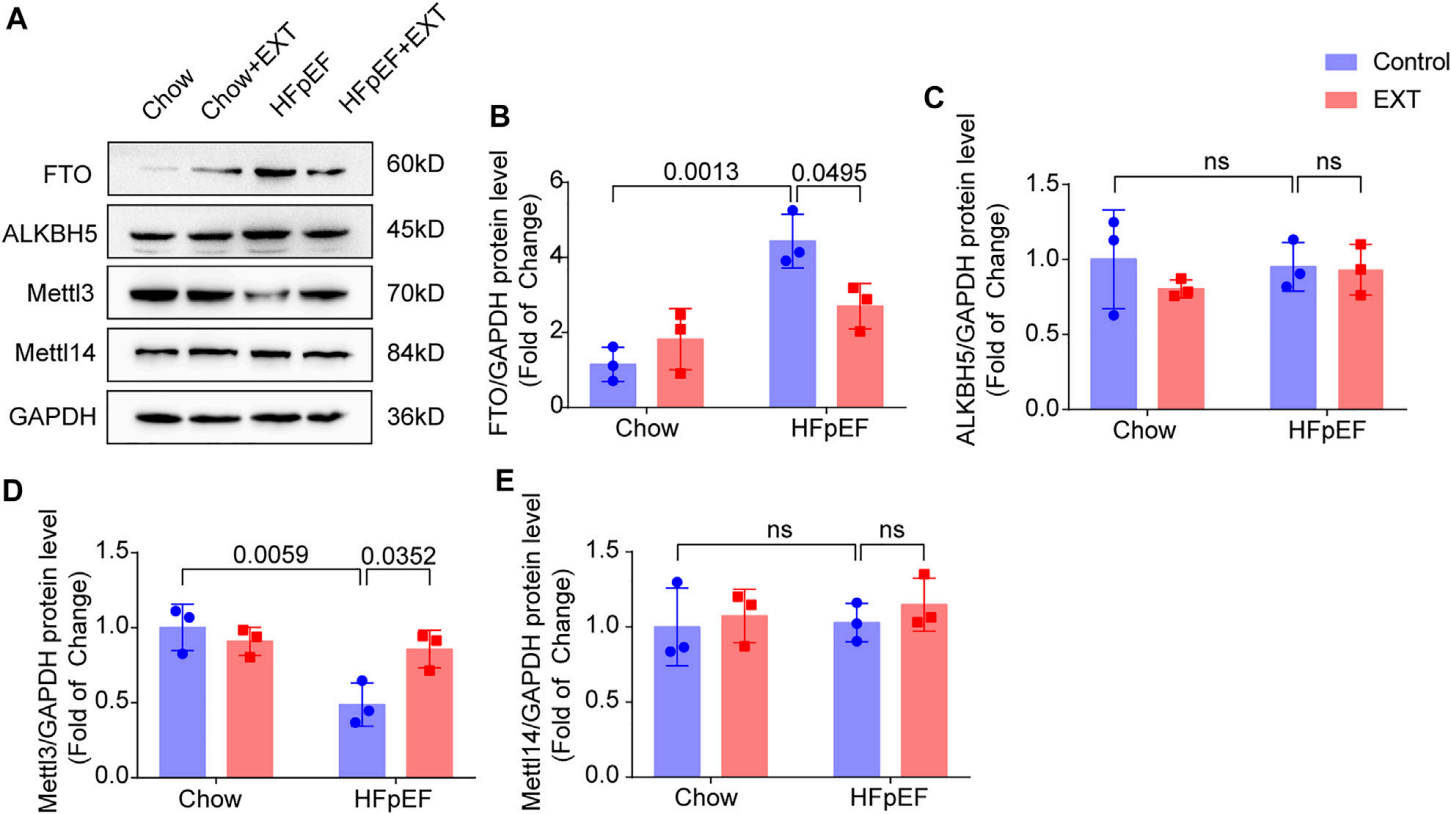
FTO is Downregulated in HFpEF + EXT compared to HFpEF. **(A–E)** Representative Western blots plots of FTO, Mettl3, ALKBH5 and Mettl14, and quantitative analysis of FTO/GAPDH, Mettl3/GAPDH protein, ALKBH5/GAPDH and Mettl14/GAPDH expressions. *n* = 3 per group. Data are presented as mean ± SEM. Two-way ANOVA and Tukey’s multiple comparison test were used. Numbers above square brackets show significant *p* values.

On basis of the analysis outcomes of unique genes in HFpEF + EXT and HFpEF hearts by GO/KEGG, we suspected that the FTO is closely related to the regulation of energy metabolism and might exert a significant effect on the pathogenesis of HFpEF. Therefore, we explored the mechanism of FTO in HFpEF through experiments. The protein level of FTO in HFpEF + EXT mice was downregulated in comparison with that in HFpEF mice. The FTO protein level detected by Western blotting was greatly lower than that in the HFpEF + EXT group in comparison with the HFpEF group (*p* = 0.001; *n* = 6 per group; Figures 5A,B). We also looked at the expression of other key methyltransferases and demethylases during m^6^A modification, including Mettl3, Mettl14, and ALKBH5. Mettl3 was increased, and no significant difference was observed in Mettl4 and ALKBH5 (Figures 5A,C–E).

**FIGURE 6:**
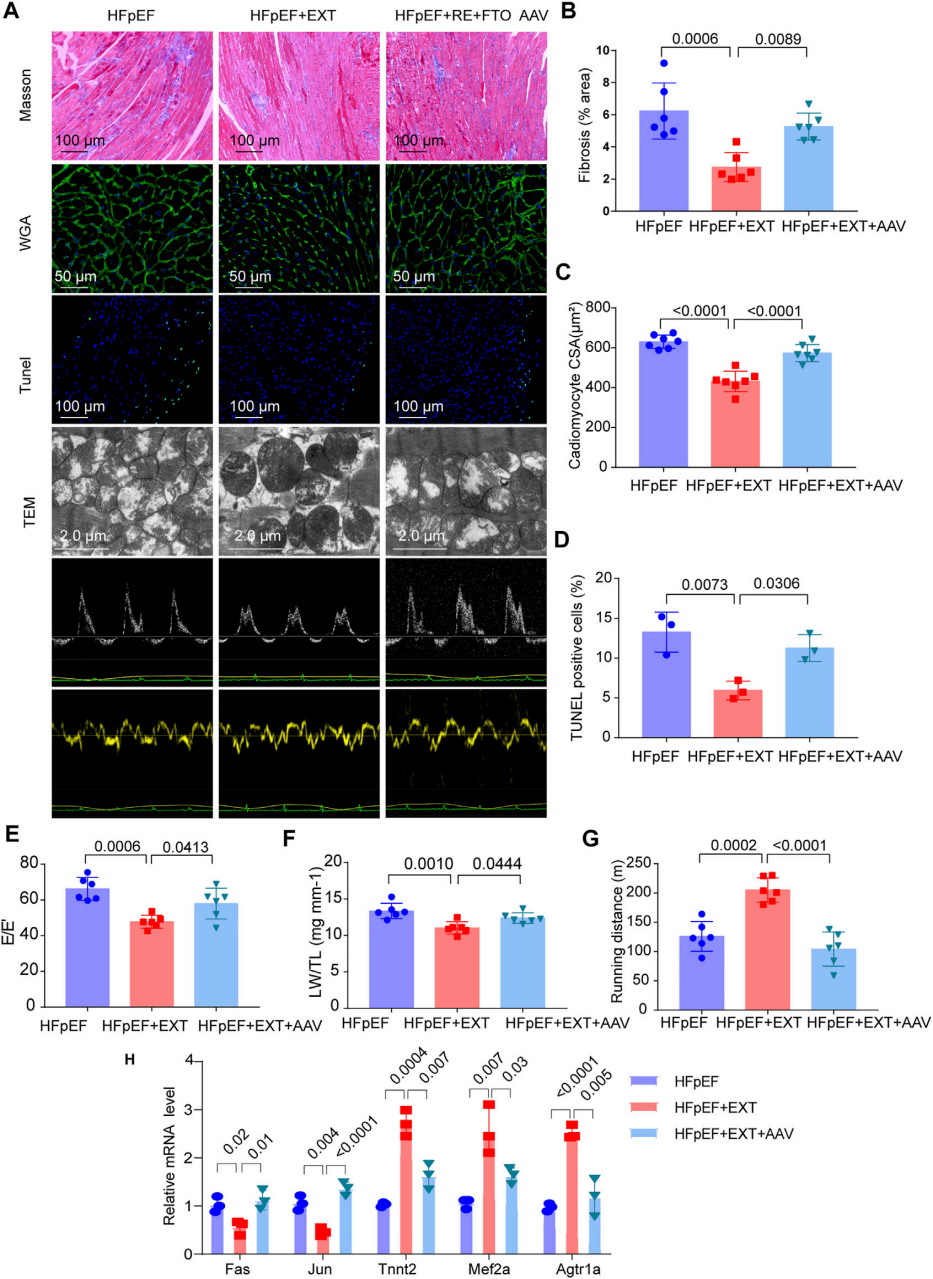
Overexpression of FTO cancels out the benefits of exercise in HFpEF + EXT mice. **(A)** Gross morphology of hearts stained with Masson, WGA, TUNEL, TEM, and Doppler echocardiography from HFpEF, HFpEF + EXT and HFpEF + EXT + AAV9 mice. **(B)** Quantitative analysis of interstitial fibrosis (*n* = 6 mice per group). **(C)** Quantitative analysis of cardiomyocyte cross-sectional area (*n* = 6 mice per group). **(D)** Quantitative analysis of TUNEL-positive cells (*n* = 3 per group). **(E)** Quantitative analysis of E/E′ ratio (*n* = 6 mice per group). **(F)** Ratio of lung weight to tibia length (*n* = 6 mice per group). **(G)** Running distance during exercise exhaustion test (*n* = 6 mice per group). **(H)** The relative mRNA levels of six genes determined by real-time PCR in HFpEF, HFpEF + EXT and HFpEF + EXT + AAV9 mice (*n* = 3 mice per group). Data are presented as mean ± SEM. One-way ANOVA and Tukey’s multiple comparison test were used. Numbers above square brackets show significant *p* values.

Overexpression of FTO cancels out the benefits of exercise in HFpEF + EXT mice by promoting myocyte apoptosis, myocardial fibrosis and myocyte hypertrophy. At the age of 16 weeks, we injected the AAV vector encoding FTO into the HFpEF + EXT mice through the tail vein, and detected the expression of FTO after 8 weeks. After injection of AAV-FTO for 8 weeks, expression of myocardial FTO increased by approximately 7.5-fold in HFpEF + EXT mouse hearts (Supplementary Figures S1A,B). After 8 weeks of adenovirus injection, we found overexpression of FTO increased interstitial fibrosis in HFpEF + EXT mouse hearts (Figures 6A,B, *p* = 0.0089). Furthermore, the overexpression of FTO efficiently augmented cardiomyocyte hypertrophy, as demonstrated by the increase in cross-sectional area of cardiomyocyte in HFpEF + EXT mouse hearts (Figures 6A,C, *p* < 0.0001). Moreover, the overexpression of FTO accelerated apoptosis in myocytes and increased the proportion of TUNEL-positive cells in the myocardium (Figures 6A,D, *p* = 0.0306). The overexpression of FTO significantly destroyed mitochondrial integrity and function by disorganized mitochondrial arrays and aggregates of swollen mitochondria with slight lysis of the cristae (Figure 5A). Furthermore, the doppler echocardiography showed that FTO overexpression exacerbated diastolic dysfunction in HFpEF + EXT mice by increasing the E/E′ ratio (Figure 6E). The overexpression of FTO reduced LW/TL and running distance (*n* = 6, *p* = 0.0444 and *p* < 0.0001; Figures 6F,G). Overexpression of FTO reduced the mRNA levels of Tnnt2, Mef2a and Agtr1a, and increased the mRNA levels of Fas and Jun (Figure 6H). Overall, these data indicated that the overexpression of FTO cancels out the benefits of exercise in HFpEF + EXT mice by promoting myocyte apoptosis, myocardial fibrosis and myocyte hypertrophy.

## Discussion

HFpEF is an aging disease characterized by cardiac hypertrophy, myocardial interstitial fibers, and cardiac diastolic dysfunction (Prandi et al., 2022). Due to inadequate understanding of the pathophysiology and animal models of HFpEF, it is difficult to develop methods to treat this more prevalent and lethal disease (Schiattarella et al., 2019; Deng et al., 2021). Unlike HFrEF, patients with HFpEF constitute a highly heterogenous population. HFpEF patients usually do not trace back to a primary lesions and injuries of cardiomyocytes but rather to a complex of series of systemic abnormalities, e.g., metabolic dysfunction, hypertension, hyperlipidemia, renal dysfunction and pulmonary dysfunction (Dunlay et al., 2017; Deng et al., 2021; Mishra and Kass, 2021). There are many reasons for the lack of effective therapies of HFpEF, but it is largely because of incompletely understanding its complex pathophysiology and the lack of appropriate animal models to study its pathogenesis. EXT is an important nonpharmacological treatment, which can reduce the risk of hospitalization and cardiovascular mortality risk (Jaconiano and Moreira-Gonçalves, 2022). Although the significance of epigenetic regulation in the control of gene expression to diagnose and treat HFpEF has been widely examined (Hamdani et al., 2021), the effect of RNA alternation on the control of gene expression to affect HFpEF has only recently been investigated. Previous studies demonstrated that the mRNA of m^6^A writers Mettl3 and Mettl4; m^6^A eraser FTO; and reader YTHDF2 are elevated in patients with HFpEF (Zhang et al., 2021a). In HFpEF mice, the mRNA of FTO and YTHDC1 is upregulated but Mettl3 is downregulated (Zhang et al., 2021a). In elderly HFpEF mice, EXT could improve exercise capacity, diastolic function, and systolic reserves and reduce pulmonary congestion (Roh et al., 2020). However, the distribution of m^6^A within transcriptome-wide in HFpEF + EXT samples is still mostly unknown. Our research showed that m^6^A RNA methylation is changed in HFpEF + EXT mice, indicating specific m^6^A alternation patterns on the transcriptome-wide and gene-specific scales that were distinct from those of in HFpEF mice. GO and KEGG analyses demonstrated that m^6^A RNA methylation differential genes between HFpEF + EXT and HFpEF mice are specifically associated with myocardial energy metabolism. HFpEF + EXT mice are observed with downregulated FTO compared with HFpEF mice, and the overexpression of FTO cancels out the benefits of exercise in HFpEF + EXT mice by promoting myocyte apoptosis, myocardial fibrosis and myocyte hypertrophy. FTO is a key m^6^A demethylase that may regulate cardiomyocyte function through catalyzing the demethylation of m^6^A on particular mRNAs, such as *Fas, Jun, Tnnt2, Mef2a* and *Agtr1a*.

Exercise modifies the m^6^A profile of HFpEF, and these differential modification genes are closely associated with myocardial energy metabolism. The pattern of m^6^A modification in HFpEF + EXT mice was different from those of HFpEF mice on both transcriptome-wide and gene-specific scales. 22 409 m^6^A peaks in HFpEF + EXT mice were determined, indicating that m^6^A is a widespread post transcriptional RNA alternation in various heart disorders. In addition, we investigated that differentially methylated m^6^A peaks in HFpEF + EXT mice are primarily present in 5′UTR, which may promote mRNA translation. Importantly, in our research, we found that in HFpEF + EXT mice, the Fas m^6^A-modified site is primarily present in the 5′UTR. Therefore, we speculated that the differential methylation of RNAs in HFpEF + EXT might affect post-transcriptional translation levels and translation efficiency of RNA. We found that genes with differential m^6^A methylation and mRNA expression in HFpEF + EXT mice were mainly enriched in processes and pathways related to myocardial energy metabolism and apoptosis pathway, such as MAPK signaling pathway, insulin signaling pathway, sphingolipid metabolism, pyruvate metabolism, apoptosis, and regulation of intrinsic apoptotic signaling pathway processes (Figures 2H,I). HFpEF is a complex disease that includes cardiac hypertrophy, myocardial fibrosis, inflammation, cardiomyocyte sarcomere disfunction and mitochondrial and metabolic defects, which lead to mitochondrial dysfunction, inflammation and myocardial cardiac diastolic dysfunction (Figtree et al., 2021; Jaconiano and Moreira-Gonçalves, 2022). Recent clinical studies found that a year of committed EXT reverses abnormal left ventricular myocardial stiffness in patients with stage B HFpEF (Hieda et al., 2021). However, because of limited access to cardiac tissue, the molecular mechanisms of HFpEF remained largely unknown. Here, our study indicated that exercise induces similar functional benefits in HFpEF mouse, and the Conjoint analysis of MeRIP-seq and RNA-Seq data revealed that exercise alters the metabolic phenotype of cardiomyocytes and improves the apoptosis of myocardial cells for HFpEF. Indeed, our findings suggested that while EXT only ameliorated the HFpEF phenotypes in this “two-hit” model, it improved the overall cardiac performance and exercise capacity. We propose that, from the mice “two-hit” model with HFpEF, EXT confers benefits. The mechanism of epigenetic regulation thus provides a new therapeutic target for HFpEF and a new approach to studying HFpEF.

Exercise improves mitochondrial function in HFpEF and affects the myocardial energy metabolism phenotype (Vega et al., 2017). Post-exercise hearts exhibited a higher capacity for fatty acid oxidation and ATP generation than non-exercise hearts in response to the stimulation of increased cardiac workload by b EXT (Moreira et al., 2020). The transcriptional coactivator PGC-1α is a core molecule in the regulation of cardiomyocyte metabolism, and it was originally recognized as a cold inducible element in mitochondrial biogenesis and necessary for the metabolic adaptations of the heart to exercise (Moreira et al., 2020). In our study, an important target gene for improving the myocardial phenotype of HFpEF during exercise is Fas. Fas ligand (FasL) is a cell surface molecule of the tumor necrosis factor family binding to its receptor Fas to induce apoptosis of Fas bearing cells (Wajant, 2002). In our study, Fas m^6^A methylation expression levels are upregulated, but the mRNA and protein expression levels are downregulated, indicating that m^6^A modification of *Fas* may influence mRNA degradation.

FTO is downregulated in HFpEF + EXT samples, and overexpression of FTO cancels out the benefits of exercise in HFpEF + EXT mice by promoting myocyte apoptosis, myocardial fibrosis and myocyte hypertrophy. In 2007, the *FTO* gene was identified in a type Ⅱ diabetes genome-wide relationship research (Dina et al., 2007). Furthermore, a population cohort research showed that the effect of FTO genes was closely associated with energy intake (Scuteri et al., 2007). However, it is not clear how FTO affects the mechanisms of obesity and energy metabolism. Our KEGG pathway analysis showed that differentially methylated RNAs are predominantly associated with the MAPK and insulin signaling pathways in cardiomyocytes. In conclusion, the FTO is critical for energy metabolism. FTO expression is downregulated in mammalian heart failure hearts and hypoxic cardiomyocytes, with higher m^6^A levels in RNA and impaired cardiomyocyte contractile performance, according to recent investigations (Mathiyalagan et al., 2019). Additionally, in mice with heart failure, the m^6^A demethylase FTO improves cardiac function *via* controlling glucose absorption and glycolysis (Zhang et al., 2021b). In this research, the overexpression of FTO deteriorates heart function by growing E/E′ and LW/TL and decreasing the running distance. Furthermore, overexpression of FTO cancels out the benefits of exercise in HFpEF + EXT mice by promoting myocyte apoptosis, myocardial fibrosis and myocyte hypertrophy.

The present study has limitations. Firstly, we did not find human heart samples to validate our experimental results. Therefore, we will next seek human heart samples to validate the important role of m^6^A modification in the pathogenesis of HFpEF. Secondly, although we found the target genes were modified by m^6^A, the mechanism of how the binding proteins control the target genes has not been explored. Next, how binding proteins affect target gene stability, translational efficiency, or degradation will be explored. Thirdly, although we demonstrated that overexpressing FTO in the heart worsened cardiac function, future research with cardiac specific knockout FTO mice and HFpEF models will investigate the actual mechanism by which FTO mediates HFpEF.

Consequently, m^6^A RNA methylation was modified in HFpEF + EXT mice, who displayed distinct m^6^A alternation patterns at the transcriptome and gene levels compared to HFpEF mice. Differentially methylated genes were found to be linked with myocardial fibrosis, myocyte hypertrophy, myocardial apoptosis, and myocardial energy metabolism in GO and KEGG studies. Exercise improved mitochondrial function in HFpEF and myocardial energy metabolism phenotype to reverse the myocardial phenotypes. FTO was downregulated in HFpEF + EXT mice compared with that in HFpEF mice, and the overexpression of FTO cancels out the benefits of exercise in HFpEF + EXT mice by promoting myocyte apoptosis, myocardial fibrosis and myocyte hypertrophy. FTO is a key m^6^A demethylase that may regulate cardiomyocyte function through catalyzing the demethylation of m^6^A on particular mRNAs, such as *Fas*, *Jun*, *Tnnt2*, *Mef2a* and *Agtr1a*.

## Data Availability

The datasets presented in this study can be found in online repositories. The names of the repository/repositories and accession number(s) can be found below: Gene Expression Omnibus accession number: GSE208354.

## Supplementary material

SUPPLEMENTARY FIGURE S1 Flow chart and adeno-associated virus overexpression FTO. . Flow chart of HFpEF modeling and running method in mice. **(B,C)** Representative Western blots and quantitative analysis of overexpression of FTO. *n* = 3 Data are presented as mean ± SEM. One-way ANOVA and Tukey’s multiple comparison test were used. Numbers above square brackets show significant *p* values.

SUPPLEMENTARY FIGURE S2 GO analysis of Differential m^6^A modification genes. Biological Process analysis of up-regulated and down-regulated m^6^A methylation genes. **(C,D)** Cellular Component analysis of up-regulated and down-regulated m^6^A methylation genes. **(E,F)** Molecular Function analysis of up-regulated and down-regulated m^6^A methylation genes.

SUPPLEMENTARY FIGURE S3 GO analysis of Differential mRNA genes. Biological Process analysis of upregulated and downregulated mRNA genes. **(C,D)** Cellular Component analysis of upregulated and downregulated m6A mRNA genes. **(E,F)** Molecular Function analysis of upregulated and downregulated mRNA genes.
