# Supplementary figures and images for: Exercise training ameliorates myocardial phenotypes in heart failure with preserved ejection fraction by changing N6-methyladenosine modification in mice model
Source: Front Cell Dev Biol. 2022 Sep 2;10:954769. doi: 10.3389/fcell.2022.954769 (PMC9478036; doi:10.3389/fcell.2022.954769)

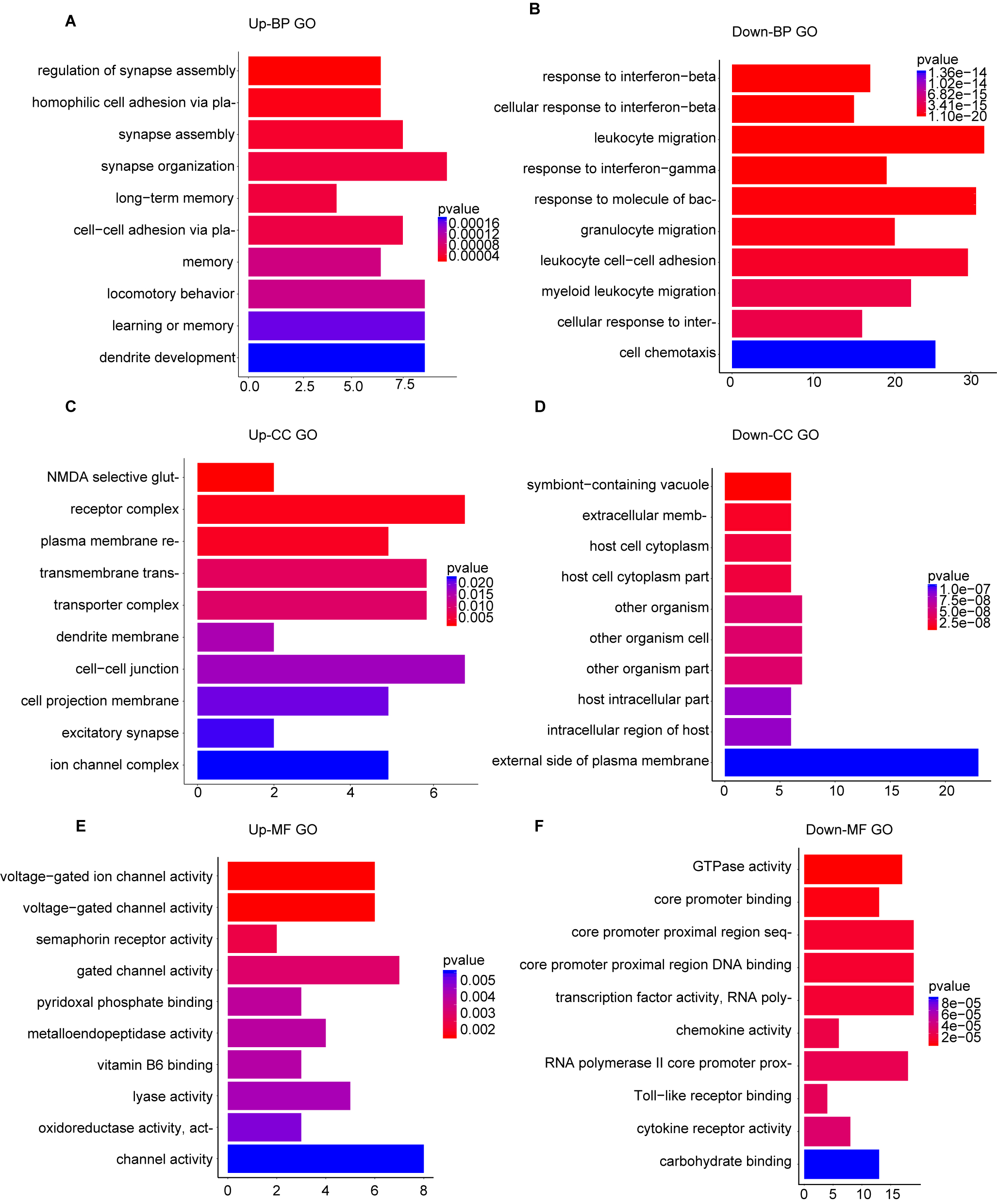

Supplement: Supplementary file 1 [file Image3.JPEG]

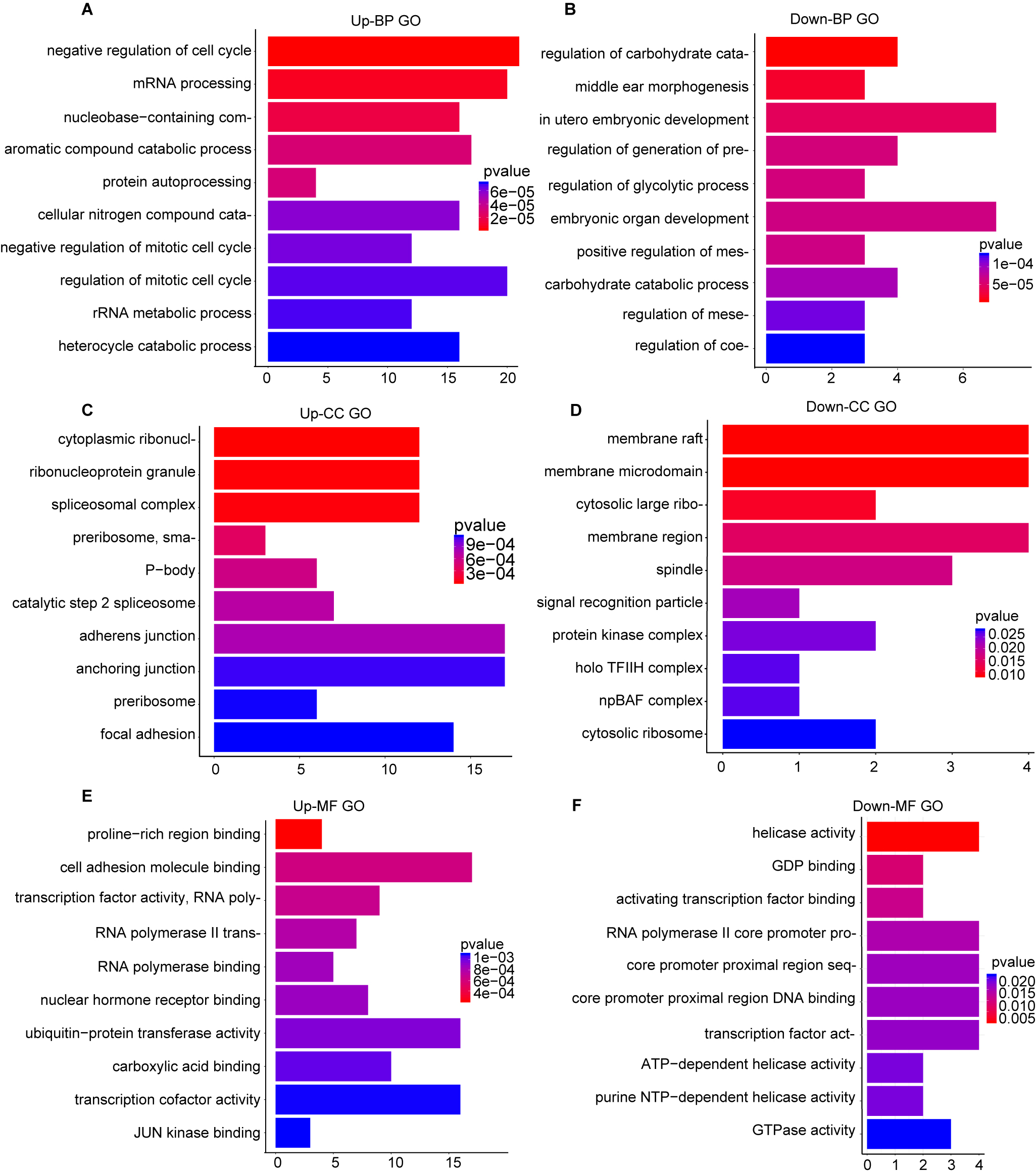

Supplement: Supplementary file 2 [file Image2.JPEG]

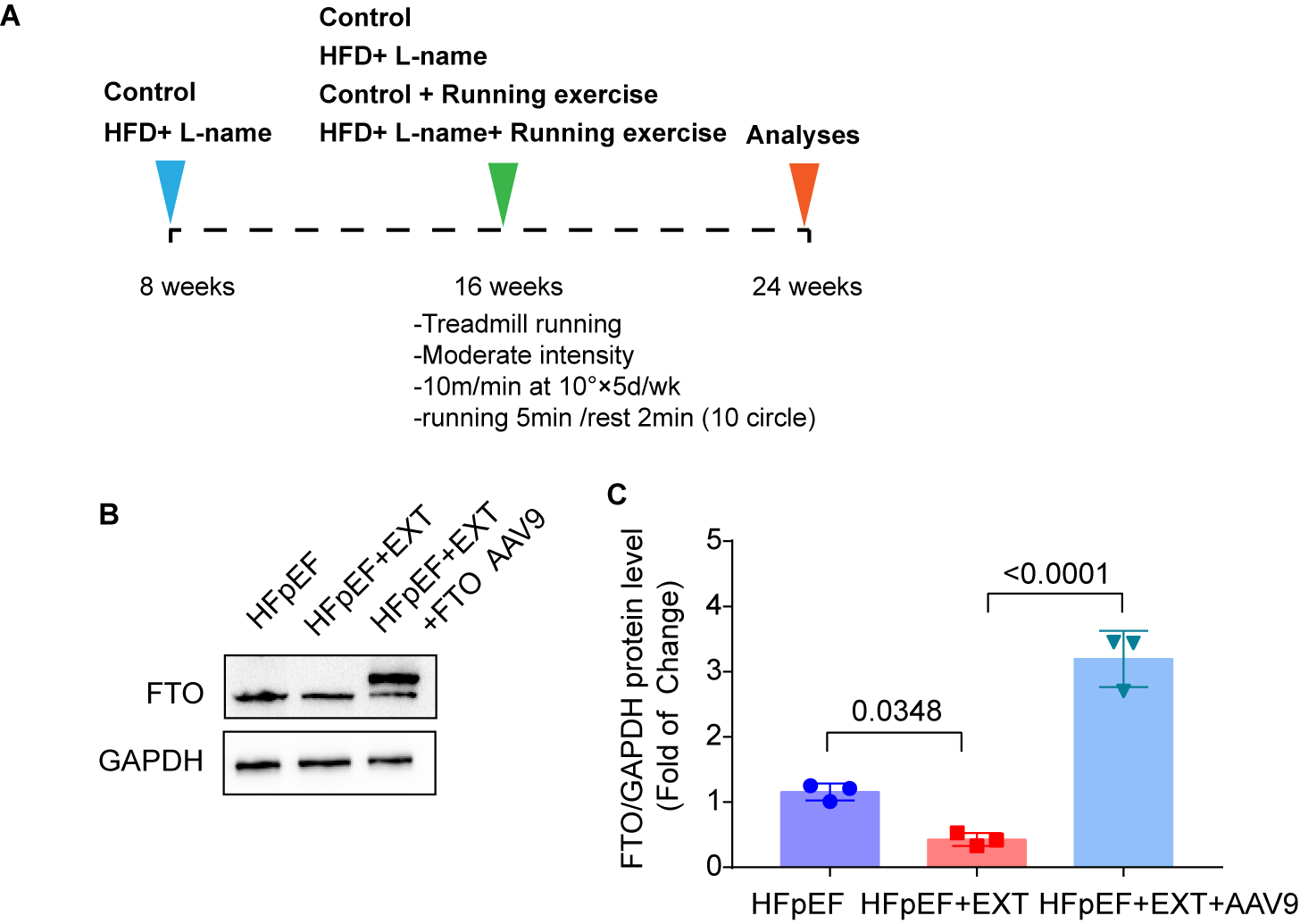

Supplement: Supplementary file 3 [file Image1.TIF]
